# Barriers and opportunities for evidence-based health service planning: the example of developing a Decision Analytic Model to plan services for sexually transmitted infections in the UK

**DOI:** 10.1186/1472-6963-12-202

**Published:** 2012-07-17

**Authors:** Catherine R H Aicken, Nigel T Armstrong, Jackie A Cassell, Neil Macdonald, Angela C Bailey, Sandra A Johnson, Catherine H Mercer

**Affiliations:** 1 Centre for Sexual Health and HIV Research, University College London, London, UK; 2 Kleijnen Systematic Reviews Ltd, York, UK; 3 Brighton and Sussex Medical School, Brighton, UK; 4 Imperial College London, St Mary’s Campus, London, UK; 5 Imperial College Healthcare NHS Trust, St Mary’s Hospital, London, UK; 6 Health Protection Agency - South East Region, London, UK

**Keywords:** Model, Decision Analytic Model, Evidence-synthesis, Service planning

## Abstract

**Background:**

Decision Analytic Models (DAMs) are established means of evidence-synthesis to differentiate between health interventions. They have mainly been used to inform clinical decisions and health technology assessment at the national level, yet could also inform local health service planning. For this, a DAM must take into account the needs of the local population, but also the needs of those planning its services.

Drawing on our experiences from stakeholder consultations, where we presented the potential utility of a DAM for planning local health services for sexually transmitted infections (STIs) in the UK, and the evidence it could use to inform decisions regarding different combinations of service provision, in terms of their costs, cost-effectiveness, and public health outcomes, we discuss the barriers perceived by stakeholders to the use of DAMs to inform service planning for local populations, including (1) a tension between individual and population perspectives; (2) reductionism; and (3) a lack of transparency regarding models, their assumptions, and the motivations of those generating models.

**Discussion:**

Technological advances, including improvements in computing capability, are facilitating the development and use of models such as DAMs for health service planning. However, given the current scepticism among many stakeholders, encouraging informed critique and promoting trust in models to aid health service planning is vital, for example by making available and explicit the methods and assumptions underlying each model, associated limitations, and the process of validation. This can be achieved by consultation and training with the intended users, and by allowing access to the workings of the models, and their underlying assumptions (e.g. via the internet), to show how they actually work.

**Summary:**

Constructive discussion and education will help build a consensus on the purposes of STI services, the need for service planning to be evidence-based, and the potential for mathematical tools like DAMs to facilitate this.

## Background

Health services consist of collections of complex clinical pathways for many patients, and as such, are challenging to plan. Compared to service delivery for individual patients, for which evidence-based approaches are well-established [1], there is less of a tradition of evidence-based service planning. For example, evidence-based guidelines exist for treating an individual presenting with certain symptoms, but it is unclear how to plan appropriate combinations of services to meet the varying needs of local populations. Even individual clinical services, such as hospitals, are often planned on the basis of limited evidence and without making assumptions explicit [2]. Planning services for infectious diseases, including sexually transmitted infections (STIs), is still more complex, since each case may or may not produce further cases [3], such that the goals of early detection and treatment are not only to improve the health of the individual but that of the wider population too [4].

Currently, evidence-based STI service planning for localities may begin with a sexual health needs assessment of the local population, typically involving collation and/or collection of (largely) quantitative data, and expert opinion, together with descriptions (or mapping) of current provision [5]. However, seldom is this information then used explicitly or directly to inform the volume of different types of services that should be provided to meet the population’s needs, often because service planners are unclear as to how this can be done [6]. Yet various tools have been used to help policy-makers choose between health technologies, and these could be developed to assist service planning in this health area. Decision Analytic Models (DAMs) are one type of tool and are well-established in Cost Effectiveness Analysis (CEA) [7] as part of Health Technology Assessment [7-10].

A DAM is a mathematical method of synthesising evidence on the outcomes and costs of alternative, mutually exclusive, healthcare interventions [10]. As an example, a simple generic DAM could be one that combined evidence on resource use (e.g. staff time) with price information (e.g. salary) to show the costs of each of the alternatives available to a decision-maker. A DAM applied to service delivery would need a more complex structure as it needs to take account of the costs of current and/or planned service provision, the features of these service provision alternatives (e.g. the numbers and characteristics of patients attending the different types of services in the locality), along with indicators of population need (e.g. demographic characteristics, disease prevalence) for public health outcomes, such as the incidence of disease. While such models are computationally demanding, increases in computing power are facilitating the consideration of such complexities.

The synthesis of different types of data is an attractive feature of a DAM, although it is important to recognise that DAMs do not act as a substitute for the best available evidence, and as a model, a DAM is only as good as the data it synthesises. Other attractive features include that a DAM’s inputs, assumptions, and model structure can all be made explicit [11], and the results of sensitivity analyses (the effect on outcomes of changes in input parameters [12,13]), can be published, revealing how the model works and how robust it is.

The MSTIC study, an abbreviation for ‘Maximising STI Control in local populations’, funded by the UK Medical Research Council (grant number G0601685) sought to develop a DAM to be used by planners of local STI services. Specifically, we aimed to develop a tool to compare the impacts of different combinations of specialist, genitourinary medicine (GUM) services and primary-care based services, on STI incidence and the associated costs and cost-effectiveness. The DAM was to be based on a model of STI transmission that took into account demographic characteristics, for example, the age-sex structure of a local population [13].

We assumed that users of the DAM would not have specialist knowledge of health economics or epidemiological modelling so we planned to present the results of the DAM as an interactive web-based tool. While several health-related web-tools are publicly available and in use (e.g. QRISK®2 [14]), they are novel in service planning [15]. We recognised that our web-tool should not require a high level of computing expertise or a steep learning-curve for its use, and so we planned that users would simply input data for their locality into the tool, with online help resources available. The web-tool would process these data using the results of the underlying model to generate outputs. By varying the data entered for different parameters, users could view the epidemiological and economic implications of changing, for example, the overall capacity or relative capacities of specialist and primary-care based services. We planned to include a technical appendix in the web-tool’s user guide that stated all the assumptions and parameter estimates used in the underlying DAM to promote transparency and, we hoped, trust in the web-tool.

To explore the extent to which our DAM, presented as a web-tool, would be considered by stakeholders as a useful and acceptable adjunct to service planning, we sought the views of service providers, public health specialists and commissioners working in sexual health. We presented our research plans for developing a DAM and web-based tool to assist the planning of STI services for local populations, as described above, to a workshop on the sources and uses of sexual health data, held in March 2009, comprising approximately 80 clinicians, public health specialists and commissioners, all with a responsibility for, or surveillance interests relating to, STI services in one region of England. Data collection was by means of notes taken during a plenary discussion, and we solicited stakeholders’ views by inviting the participants to discuss the tool with us after the discussion (face-to-face or by email after the event), and provided sticky notes to be stuck onto a flip-chart – conduits for public and more private feedback. In this paper, we describe the themes that emerged rather than present direct quotations, as we did not request participants’ permission to quote them, nor did we record the workshop.

Here we present the major themes expressed that were of interest and concern for the stakeholders. We then discuss implications for the development, dissemination and potential use of DAMs in the planning of local health services.

## Discussion

First we present reactions emerging from our initial presentation of our research plans. We then discuss possible reasons for these, and considerations for others attempting similar work in this area.

### Reactions and responses to the concept of a DAM for STI service planning

Views expressed in the plenary were generally sceptical about the relevance and utility of a DAM for STI service-planning. However, following this discussion several individuals privately expressed support for our proposed DAM and web-tool and offered constructive feedback. Three key themes emerged from the workshop:

1) Tension between the individual and population perspectivesA number of stakeholders expressed a focus on the individual patient’s health benefit, and more generally the patient’s healthcare *experience*, almost to the exclusion of wider public health benefit that services might provide, i.e. their impact upon rates of infection. As such, some interpreted the MSTIC study’s objective as directly opposed to providing ‘holistic’ patient-centred care, and criticised the DAM’s lack of assessment of more qualitative aspects of healthcare provision because of its focus solely on quantifiable inputs and outputs. Given this perceived conflict between the individual and population perspectives, it was unsurprising that some stakeholders were wary of cost-effectiveness evidence as well as health economics more generally.

2) ReductionismThe proposed DAM was criticised for being ‘reductionist’, and some stakeholders argued that it overlooked the complexity of service provision and the ‘real world’. For example, some felt that the DAM’s consideration of the different types of STI service that may be available in a locality was over-simplified and that it did not capture the variability in service provision, for instance between different GUM clinics and between different general practices.The ‘reductionist’ criticism also applied to the DAM’s inputs and outputs. In our presentation, we explained that in a DAM’s development, inputs can be varied within realistic ranges in order to determine which are most influential on the outputs (sensitivity analyses) [11,12] and conversely which data inputs are relatively unimportant and so need not be collected. We thought that the volume of information service planners can reasonably be expected to gather, as well as the ease with which they can do so, would be important considerations. Consequently, we perceived the reduced burden of data collation associated with using a DAM as an attractive feature. However, a number of the audience interpreted the concept of making decisions based on a reduced set of variables as *less* evidence-based. Indeed, some participants later in the event called for *more* data collection to inform improved service planning.

3) Lack of transparencyFormulation of a DAM involves building a structure that synthesises key inputs, which, as we explained to the audience, we planned to present as an interactive web-tool to make the DAM easily accessible. We perceived this as an additional strength of our model. However, our audience was concerned that the DAM would be a ‘black box’ and called for the model’s workings not to be concealed.Our perceived motives for developing a DAM to assist in STI service planning were also a source of criticism. For example, our plan to incorporate cost-effectiveness evidence into our model was viewed by some as an underhand threat of service cuts, or as providing evidence that could be exploited either for that purpose or to redistribute resources between services. However, we observed a public/private split in expressed attitudes to the proposed DAM, as several participants approached us privately later in the event expressing support for the use of health economic methods in service planning and engaged in discussion about the assumptions a DAM could reasonably make.

### Why are quantitative, model-based tools for service planning helpful? And why are they not more popular?

The increasing diversity of health services providing care for STIs in the UK [16], together with the dynamic nature of STI transmission [3], make it increasingly important to assess the impact of different combinations of service provision with varying clinical outcomes on STI control in local populations. While tools based on such quantitative evidence can be used to make explicit comparisons between service configurations for a locality, our consultation found that tools like a DAM may be perceived as threatening and reductionist by clinicians, local public health staff and service commissioners. This may reflect epistemological differences in the ways in which we, as academic researchers, and our stakeholders approach STI service planning, including what constitutes valid evidence, and how this evidence should be brought together. An example of this difference was the particular tension we observed between the individual and public health perspectives, reflecting the differing goals of the clinician and public health advocate [17]. Another example is a misunderstanding of the use of health economics, as others have discussed [18], which may in part reflect cynicism in the way the language of economics is perceived to be used by public figures attempting to justify cost-cutting, and not simply a lack of understanding of the discipline.

Lack of understanding of models may contribute to health professionals’ reluctance to follow their recommendations [19], and this can apply to DAMs as well as other mathematical models. Furthermore, where a tool originates outside a user’s discipline, and he or she may not know (or trust) the interests of those who created and funded the model, this can also contribute to mistrust in the results [20]. Yearley [20] reported that public understanding of computerised scientific models depends on the trustworthiness of the institutions developing models, the public’s own knowledge (and lack of recognition by scientists of this), and the evaluation of assumptions within models (which may indeed not be appropriate), which can override the credibility and appeal that models might otherwise have. As publicly-funded scientists working on the MSTIC study, we must clearly emphasise to potential users of our web-tool that we have no stake in the cutting or funding of particular types of service – but it remains unclear how much reassurance our separation from decision-making will give.

As noted above, assumptions made and data used by a DAM can limit its validity – yet these potential limitations can apply to other methods for planning services too. Where DAMs (and other mathematical models) contain parameters which are poorly understood and therefore particularly uncertain, for example, transmission probabilities, then different models of the same outcome can result in very different predictions, as was the case with predicting the population impact of chlamydia screening [9,21-23]. Together with the media’s coverage of models that have been less successful in their predictions (e.g. climate change modelling, swine flu modelling) [24,25], this is likely to exacerbate health professionals’, and more generally, the public’s mistrust in the validity and utility of modelling. Further robust validation of tools such as DAMs for service planning, in any case a valuable exercise, may enable us to advocate more confidently their use in this area, and perhaps allay some of the scepticism of clinicians and commissioners. Nevertheless, among those whose reservations about DAMs for service planning are more fundamental (mistrust in health economics and/or a quantitative approach to planning, for example), it is unclear whether further validation will be convincing.

We note that DAMs do not constitute ‘research evidence’ in the empiricist tradition (such as the results of randomised controlled trials or service evaluations). Neither do they ‘fit’ within a conventional step in the commissioning cycle [26]. Unsurprisingly, the UK’s National Institute for Health and Clinical Excellence (NICE) has produced guidance informing the commissioning of services for various types of cancer [27], but not for STIs or indeed any infectious diseases. The potential role of DAMs is, as we have seen, untested and their use under-developed at the present time and, as discussed earlier, less straightforward for infectious diseases. The relative novelty of using models for STI service planning may therefore have made it difficult for stakeholders to engage with, and discuss, our proposed DAM and web-tool.

There is however a clear need to improve evidence-based service planning as current efforts involve collation (and often collection) of a considerable amount of data, combined in ways which may be neither transparent nor, in effect, any less reductionist. This is not only time-consuming but there is also evidence of a large duplication of work between those planning services for different localities in the same health areas [28]. It was therefore interesting to see that our proposal to use a DAM, which would reduce the amount of data required for evidence-based planning by identifying only key parameters, was unfavourably received in our consultation. On the contrary, there were calls for more data collection rather than evidence-based methods of using existing data.

The difference we observed between publicly and privately expressed views on the DAM is telling, and may reflect tensions between roles, particularly between clinicians and those in managerial/planning roles [29]. While ultimately it is commissioners of local services, within budgetary and other constraints, who must decide which combination of services to fund [28], there is a need to acknowledge the objectives, expertise and role that clinicians and public health leads play. This though can create conflict when commissioners consult local experts in sexual health while also considering cutting or expanding these individuals’ services. Explicit comparisons of cost-effectiveness when assessing the public health impact of services, for example, through using a DAM, may raise awkward questions to the different parties in these collaborations, particularly around funding allocation. Central to evidence-based planning of service delivery is therefore the creation and nurturing of meaningful dialogue among clinicians and managers [30], together with commissioners. This will be increasingly important given changes to the commissioning of health services specified in the *Liberating the NHS* White Paper [31], as well as in the context of public spending cuts.

## Summary

It is apparent that there is a need for more and better dialogue between health professionals on the one hand, and epidemiologists and health economists on the other, to improve understanding of the benefits and limitations of mathematical decision-making tools such as DAMs. For example, while our approach was perceived by some stakeholders as reductionist, the fact needs to be conveyed that all service planning methods make simplifications and assumptions, and are reliant on the accuracy of the available data. Simplifications are not always justified or even made explicit, so that detailed critique and discussion is lacking. Ongoing consultation is therefore essential for the development and implementation of novel technology and methods. With an increasing emphasis in publicly funded research on how research feeds into public policy and practice [32], this is an important skill for public health researchers to develop.

Of equal importance is the need for continued learning among clinicians and decision-makers regarding the nature and value of evidence and methods from different disciplines. Researchers can clearly play a part in this as well, for example, by using innovative methods for explaining health economic methods and cost-effectiveness (e.g. Democs [33]), and not only by demonstrating the validity of DAMs for service planning but by communicating how validation has been undertaken. We also believe that allowing users easier access, such as through our web-tool, is vital if DAMs are to be transparent. Thus, constructive discussion and education will help build a consensus on the purposes of STI services, the need for service planning to be evidence-based, and the potential for mathematical tools like DAMs to facilitate this.

## Abbreviations

DAM: Decision Analytic Model; MSTIC: Maximising Sexually Transmitted Infection Control in local populations; STI: Sexually Transmitted Infection; UK: United Kingdom of Great Britain and Northern Ireland.

## Competing interests

The authors declare that they have no competing interests.

## Authors’ contributions

The paper was conceived by CA, NA, JC, AB, SJ and CM at an event organised and co-ordinated by SJ. All authors were present at at least one of the stakeholder events. CA led in writing the first draft, with substantial input from NA, and all authors contributed to the writing of the manuscript. All authors read and approved the final manuscript.

## Authors’ information

CM was the Principal Investigator of the MSTIC study, CA was the Study Co-ordinator, and NA, JC and NM members of the MSTIC study team. AB and SJ are collaborators of the MSTIC study.

## Pre-publication history

The pre-publication history for this paper can be accessed here:

http://www.biomedcentral.com/1472-6963/12/202/prepub
